# 3D simulation of radiographic projections to test and reduce the effect of pelvic tilt on the accuracy of cross-table lateral radiography

**DOI:** 10.1186/s12891-020-03858-2

**Published:** 2020-12-14

**Authors:** Jie Xu, Baohua Su, Wenhui Zhang, Hao Sun, Deng Li, Zhiqing Cai, Meiyi Chen, Meiling Qiu, Ruofan Ma

**Affiliations:** 1 Department of Joint Surgery, Sun Yat-sen Memorial Hospital, Sun Yat-sen University, Guangzhou, China; 2 Department of Orthopedic Surgery, the Third Affiliated Hospital of Guangzhou Medical University, Guangzhou, China

**Keywords:** Version angle, Acetabular component, Cross-table lateral radiography, Total hip arthroplasty, Pelvic tilt

## Abstract

**Background:**

Cross-table lateral (CL) radiography is a convenient and feasible method to assess cup version angle (VA) after total hip arthroplasty; However, pelvic tilt (PT) may contribute to its measurement inaccuracy. How PT affects CL radiographic measurements have not been well studied. We sought (1) to determine the effect of the PT on cup version measurement on CL radiography and (2) to develop a method for reducing measurement errors caused by the PT.

**Methods:**

We used 3D technique to construct standard model and capture CL radiography simulation. A linear regression model was created to analyze the relationship between PT and VA. CL radiography and computed tomography (CT) were performed for the enrolled patients after surgery. The consistency between CL and CT measurements were verified by intra-class correlation coefficient (ICC).

**Results:**

There was a high correlation between the VA and PT. For each 1-degree increased in the PT, the VA decreased by 0.76° (R^2^ = 0.995, *p* < 0.001). Based on the data, we created a corrective formula to convert the radiographic measurements into values approximating the actual VA under a natural pelvic position. The VA measurements corrected by our equation was in high agreement with the CT-measured values with reference to the corresponding PT (ICC = 0.988, *p* < 0.001), which was in sharp contrast to that without PT control (ICC = 0.454, *p* = 0.203).

**Conclusions:**

The PT may contribute to cup version measurement inaccuracies on CL radiography. Our mathematical algorithm can serve as a reliable method to improve the accuracy of CL radiography.

## Background

Malposition of acetabular components during total hip arthroplasty (THA) is known as the major risk factor contributing to complications, such as dislocation [1], impingement [2], and accelerated bearing wear [3]. Accurate assessment of the version angle (VA) of acetabular components is important to predict the potential risk of these complications after THA [1–5]. Computed tomography (CT) is considered the most accurate method for the measurement of cup anteversion; however, cost burden and radiation exposure may limit its application on a routine basis [6–8]. Conversely, plain radiography is still commonly employed in clinical practice, including anteroposterior (AP) radiography and cross-table lateral (CL) radiography.

CL radiography may be a more convenient and feasible method than AP radiography, in which cup version can be directly measured and anteversion and retroversion can be discriminated from each other [9, 10]. Although it may provide acceptable assessment of acetabular component position, it is not reliable enough for precise measurement [10–12]. The relationship between the pelvic tilt (PT) and cup version measurement is well established. Previous studies have consistently shown that each degree of anterior or posterior tilt of the pelvis will change the cup version measurement by approximately 0.8° [13–16]. Concerns on the influence of pelvic inclination variations caused by flexion of the contralateral hip joint during CL film shooting on the accuracy of cup version measurements have been raised. However, the extent to which the PT affects CL radiographic measurements and how to compensate for this effect have not been well studied.

In this study, we aimed to determine the effect of the PT on cup version measurements on CL radiography and to develop a feasible method for modifying measurement inaccuracies caused by the PT.

## Patients and methods

### 3D model building

**Fig. 1:**
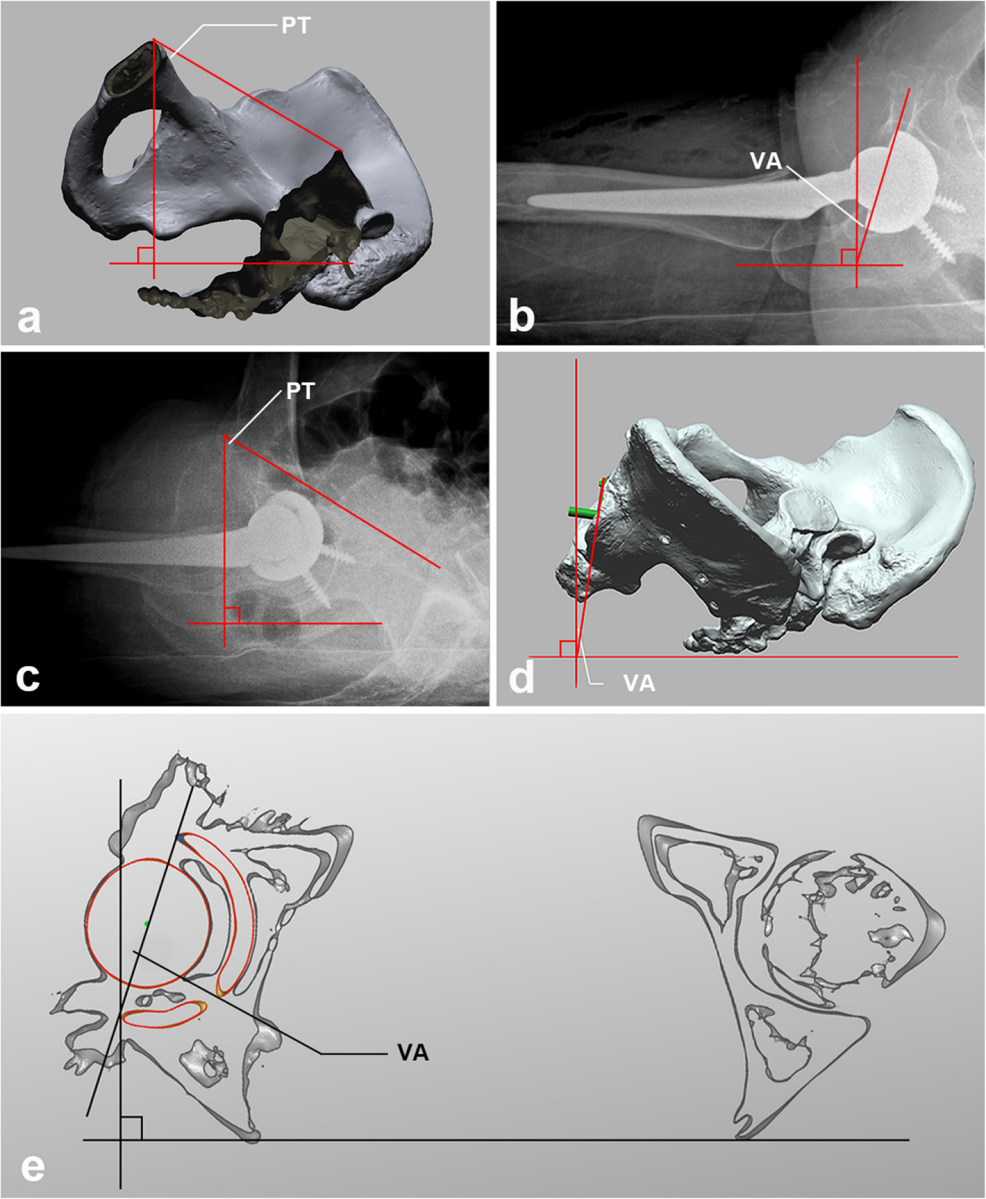
**a** Pelvic Tilt in 3D model. Pelvic tilt = the angle created by a line running from the first sacral midpoint to the symphysis pubis and a line perpendicular to the horizontal plane in the supine position. The normal value is about 60°. **b** Measurement of VA on Cross-table lateral Radiography. VA was measured using the following formula: anteversion = angle between a line along the rim of the cup and a line perpendicular to the horizontal plane. **c** Measurement of pelvic tilt from the lateral radiograph. **d** VA Measurements in 3D Simulation. Cross-table radiography was simulated by capturing a screenshot in the lateral position with rotation angles of 45°. PS. As the edge of the cup was obscured by the acetabulum, we reconstructed an acetabulum axis (the green component in the figure) for convenience and accuracy of measurement. **e** VA measurement on the transverse plane of 3D-transformed and PT-adjusted CT with the reference of posterior aspects of the ischium

To identify the influence of the PT on the acetabular cup version measurement on CL radiography, we constructed the 3D postoperative models of the pelvis using Geomagic Design X 2016 (Geomagic Inc., Morrisville, North Carolina, USA). Initially, we used a laser equipment to scan the titanium converge acetabular cups with a diameter of 48–52 mm (Trilogy® Acetabular Hip System, Zimmer, Warsaw, Indiana, USA) to establish the cup models. A CT scan for pelvis was performed on three healthy female volunteers without hip deformities and previous surgeries. We defined the PT as the angle created by a line running from the first sacral midpoint to the symphysis pubis and a line perpendicular to the horizontal plane in the supine position (Fig. 1a). Normal value is about 50° ~ 60°. An inclination of 60° was set as the natural position of the pelvic models. According to the safe zone described by Lewinnek et al. [1], an anteversion of 15° and an inclination of 45° were set as the initial position before the cup models were implanted. After the parameters were settled down, the postoperative hip model was completed by implanting the prostheses into the pelves. On this model, parameters including VA and PT can be set as different unit by intervals of 5° independently.

### Patients

A total of 50 patients who underwent primary THA for hip degeneration diseases were recruited in this study between July 2019 and December 2019, including 21 men and 29 women with an average age of 56 years (range, 32–78 years) and body mass index of 22.74 kg/m^2^ (range, 17.60–24.84 kg/m^2^). None of the patients had spinal or pelvic surgeries or deformities. The same team of surgeons performed all cementless THAs using a posterolateral approach or a direct anterior approach. All acetabular cups were selected from the Trilogy® Acetabular Hip System and R3® Acetabular Hip System (Smith & Nephew, Inc., Memphis, Tennessee, USA). This study was approved by the ethics committee of our institution. All patients provided written informed consent.

### VA and PT measurement on CL radiography

The method of CL radiography has been described in detail in a previous study [17]. The patients were placed in the supine position with the contralateral hip flexed at 90° and the contralateral leg placed on a small stand to maintain the position. The radiation beam was positioned parallel to the examination table at 45° to the long axis of the body, and the radiographic film was positioned perpendicular to the examination table. We performed CL radiography 1 day after surgery for each patient and measure VA using the method introduced by Woo and Morrey [9] (Fig. 1b). After CL radiography was performed, we adjusted the X-ray incidence from 45° to 90° to enable the pelvic landmarks displayed completely. Then an additional lateral radiography was obtained to measure the PT. The method used to measure the PT on lateral radiograph was based on the PT definition (Fig. 1c).

To simulate VA measurement on 3D model, we placed 3D model in the supine position, rotated it clockwise to 45°, and centered it on the top of the acetabular cup. Thereafter, the model was projected onto the coronal plane, and a two-dimensional image was captured by screen as a CL film simulation. VA was measured on 3D simulated CL radiographs under different setting values of PT and VA (Fig. 1d).

### Consistency verification by CT

The accuracy and reliability of the measured VA was verified by compared with the CT value. The patients underwent a CT scan for the pelvis 7 days after surgery and were placed in the supine position with the bilateral hip joints in a neutral position. To ensure that the VA measured on CT and CL radiography was compared at a consistent PT, we used Geomagic Wrap 2017 (Geomagic Inc.) to create the 3D pelvic model based on CT data so that the PT could be set to a certain value. The VA was measured under the PT set to the measured value on the radiographs to compare the CL radiography-measured VA. The method of measuring the VA on CT was referenced to a previous study (Fig. 1e) [10].

### Statistical analysis

Three qualified orthopedic surgeons performed all measurements. The intra-class correlation coefficient (ICC) and 95% confidence interval were calculated to assess the intra- and inter-observer reliabilities. We used the two-way random effects intra-class correlation model and absolute agreement to calculate the ICC: an ICC of 1 indicated perfect reliability, while an ICC of 0 indicated the opposite. Linear regression was used to assess the correlation between the PT and acetabular cup anteversion. The ICC was used to determine the validity of the measured VA and corrected VA by comparing them with the corresponding CT values. Statistical analyses were performed using SPSS ver. 22.0 (SPSS Inc., Chicago, Illinois, USA). The significance level was set at *p*-values of < 0.05.

## Results

### Repeatability

All intra-observer and inter-observer ICCs were excellent for the measurements of the PT obtained from the additional lateral radiographs and VA obtained from the CL radiographs, 3D-simulated CL radiographs, and CT scans (Table 1).

**Table 1:** Reliability of All Measurements

	ICC for Intra-Observer (95% CI)	ICC for Inter-Observer (95% CI)
PT	0.962 (0.946 to 0.987)	0.933 (0.899 to 0.963)
VCL	0.938 (0.891 to 0.963)	0.923 (0.897 to 0.941)
VCL_3D_	0.953 (0.935 to 0.968)	0.962 (0.946 to 0.987)
VCT_0_	0.982 (0.968 to 0.989)	0.936 (0.916 to 0.958)
VCT_1_	0.948 (0.968 to 0.989)	0.925 (0.889 to 0.953)

ICC, intraclass correlation coefficient. CI, 95% confidence interval. Mean (95% confidence interval). PT, pelvic tilt measured on lateral radiographs. VCL, version angle measured on CL radiographs. VCL_3D_, version angle measured on 3-D simulated CL radiographs under different actual VA and PT. VCT_0_, version angle measured on CT without adjusted the pelvic tilt. VCT_1_, version angle measured on CT which was converted into 3-D model and adjusted PT to the value measured on radiographs

### Effect of the PT on VA measurement

**Fig. 2:**
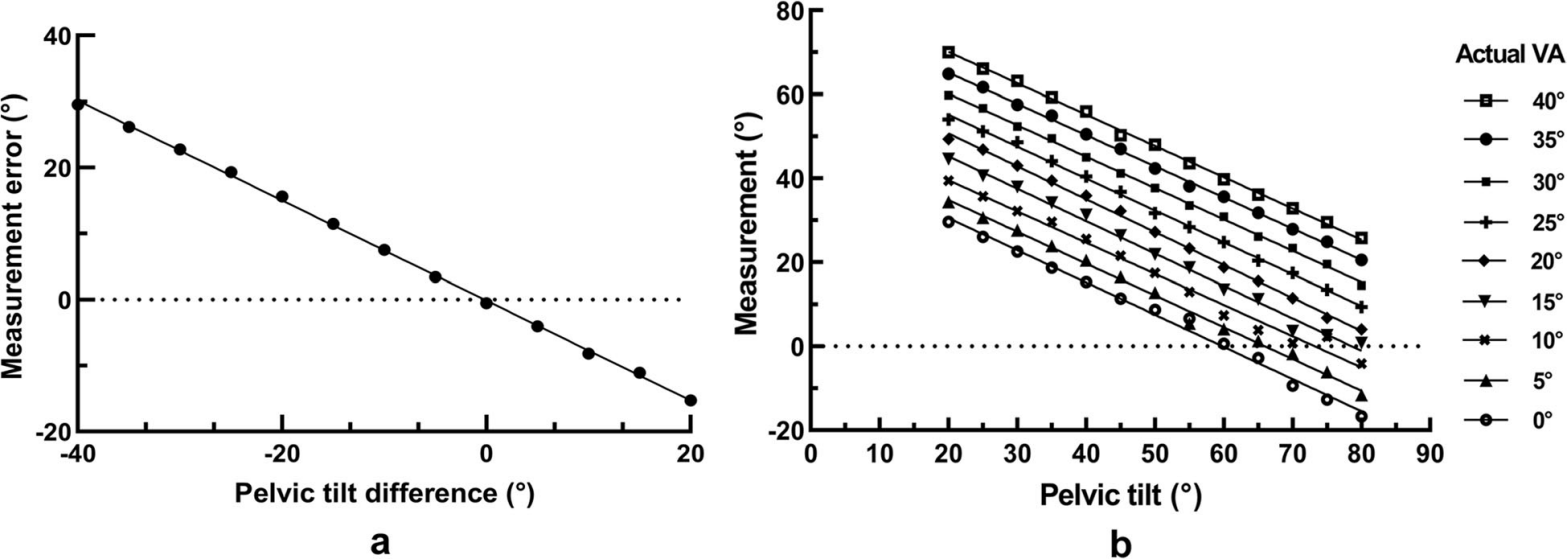
**a** Linear correlation between pelvic tilt difference and measurement error. The linear regression equation was *y* = − 0.76*x* − 0.13 (R^2^ = 0.995, *p* < 0.001). Pelvic tilt difference = Setting pelvic tilt− 60°, Measured Error = Measurement−Actual Anteversion. **b** Linear correlations involving pelvic tilt and measurement under different actual VAs

The mean values of the VA under different actual anteversion angles and PTs are shown in Table 2. A significant tendency could be seen from the scatter plot (Fig. 2a) drawn on the basis of the measured error under the PT difference. The measured error decreased along with the PT closer to 60°. A Pearson correlation coefficient of 0.998 (*p* < 0.001) was obtained in the correlation analysis, indicating a high correlation between the measured error and PT. The linear regression equation was *y* = − 0.76*x* − 0.13 (R^2^ = 0.995, *p* < 0.001), showing that for each 1-degree difference in the PT from the standard value (60°), the measured error increased (0.76°).

**Table 2:** Mean measured values under different PTs & actual VAs

Pelvic Tilt	Actual VA								
	0°	5°	10°	15°	20°	25°	30°	35°	40°
20°	29.60	34.41	39.44	44.59	49.34	54.06	59.82	64.84	70.05
25°	26.03	30.62	35.71	40.65	46.87	51.18	56.69	61.68	66.16
30°	22.57	27.63	32.17	37.99	43.02	48.65	52.39	57.50	63.23
35°	18.70	23.90	29.60	34.20	39.42	44.09	49.58	54.92	59.33
40°	15.30	20.49	25.53	31.32	35.86	40.44	45.00	50.54	55.93
45°	11.39	16.54	21.55	26.41	32.22	36.80	41.22	47.02	50.26
50°	8.68	12.83	17.48	21.84	27.24	31.73	37.69	42.36	48.03
55°	6.66	5.48	12.98	18.72	23.28	28.39	33.58	38.12	43.66
60°	0.66	4.11	7.34	13.45	18.76	24.79	30.86	35.59	39.83
65°	−2.76	1.32	3.84	11.21	15.51	20.37	26.16	31.80	36.12
70°	−9.40	−1.83	0.82	3.59	11.45	17.47	23.34	27.89	32.87
75°	−12.75	−6.13	2.31	2.64	6.78	13.46	19.55	24.85	29.52
80°	−16.70	− 11.63	−4.16	0.80	4.05	9.41	14.44	20.55	25.81

### Equation derivation procedure

From the data presented in Table 2, different linear correlations between the PT and measurement under the different actual VAs are shown in Fig. 2b. By integrating the different linear correlations, we used eq. 1 to represent the gross linear correlations between the PT and measurement; b was defined as the intercept on the y-axis. We established a mathematical correlation (eq. 2) between the intercept of the lines representing the different actual VAs on the y-axis and the corresponding actual VA. Finally, the two equations were integrated and transformed to obtain eq. 3, which could be applied to convert the VA into the actual value in the 3D-simulated CL radiograph under the PT of 60°.

1 $$ V{A}_m=0.76\times PT+b $$

2 $$ b=0.99\times VA+45.49 $$

3 $$ VA=\frac{0.76\times PT+V{A}_m-45.49}{0.99} $$

*VA*_*m*_*: Measurement of the acetabular cup version angle. VA: Actual acetabular cup version angle set in three-dimensional simulation. b: y-intercept*

### Accuracy and agreement

The consistency test indicated that both the measured value of the VA (ICC = 0.997, *p* < 0.001) and its formula-converted value (ICC = 0.988, *p* < 0.001) were in high agreement with the CT-measured value adjusted by the corresponding PT. In contrast, the consistency between the anteversion measurement and CT measurement without PT adjustment was poor (ICC = 0.454, *p* = 0.203).

## Discussion

In the present study, we established 3D postoperative models for CL radiograph simulation and data analysis. The advantages are that each variable can be set independently within a wide range, and confounding factors can be effectively controlled. The analysis showed that the PT had a significant effect on the measurement of the VA on CL radiographs. The formula we derived can convert the measured values under different PTs into actual values under the natural pelvic position. After comparing the corrected values from the clinical measurement data with the CT values, we found that they were highly consistent, which confirmed the high fidelity and reliability of the 3D simulation method and inferred formula. Our correction approach can effectively improve the accuracy of CL radiography when measuring the VA.

This study has several limitations. First, the data in our study were obtained from 3D models based on cases of a non-deformed pelvis because we sought to avoid the interference of other confounding factors (such as osteophytes and deformities) when determining the effects of the PT. The findings may not be suitable for abnormal cases. Second, we used the outline of the 3D images to replace the actual radiographs, enabling the setting of variables to be more accurate and extensive; however, the magnification of the radiographic edge cannot be well simulated, although it will not have a great impact on the measurement of the central visual field.

Reports in the literature with regard to the accuracy of CL radiographic measurement compared with that of CT measurement are conflicting [6, 11, 18, 19]. Some investigators found that the CL radiographic value was smaller than the CT value [11, 19], while others revealed the opposite [6, 18]. However, no control or influencing factors, such as the PT, was examined in those studies. The current study bridged the gap and identified the significant implications of the PT on the accuracy of CL radiographic measurement. When the PT changed by 1°, the measurement error could reach 0.76°. If the PT deviates significantly from the norm, the error may be considerable. Unfortunately, during the course of CL film imaging, the flexion of the lower contralateral extremities to avoid occlusion would lead to variations in the PT, which would be more pronounced in patients with contralateral hip stiffness. Moreover, for CT measurement, owing to the factors of anatomical specificity, pathological changes, and poor posture, there will inevitably be potential differences in the PT among different patients. When the PT was balanced between the CL radiographic and CT measurements, we were pleasantly surprised to find that the two measurements were highly consistent (ICC = 0.997, *p* < 0.001); however, these were in sharp contrast to those without control (ICC = 0.454, *p* = 0.203). Accordingly, we believe that the PT is the main factor affecting the accuracy of CL radiographic measurement.

To reduce this effect, we derived a correction formula, which can convert the measured values under different PTs into the actual VA values under the natural pelvis, and it was verified to be reliable after comparison with the CT values. Several methods have been described for correcting the error of VA measurement owing to the PT in previous studies [15, 20, 21]. Some of them used formulae as we did [15, 20], while others modified methods using special techniques, such as normograms [21]. However, these methods are relatively complex and cumbersome. In contrast, our method can achieve reliable correction only through a simple equation.

Nevertheless, both the definition of the safety zone and the correction method were evaluated on the basis of the supine position. It has been reported that there are different changes in acetabular cup anteversion in different positions. Lazennec et al. [22] performed a CT scan in the supine and standing positions in patients undergoing hip replacement, finding that the VA significantly increased in the standing position because of the change in the included angle between the patient functional plane and the APP, which exceeded the limits of the safe zone defined by Lewinnek et al. [1]. Therefore, only evaluating the acetabular component anteversion in the supine position is not sufficient. Mathematical algorithm may be employed to solve this problem. For instance, a patient was selected to receive CL radiography and additional lateral radiography to measure the VA and PT in the supine position 1 day after THA. One more lateral radiography at the standing position was performed on this patient to measure the PT 1 month after surgery. Through our equation, the actual VA in the standing position was calculated. If we include a few more patients for analysis, a new formula between the PT and VA in the standing position would be developed, indicating that by obtaining only a lateral film to measure the PT in the standing position, we can determine the actual VA using the equation. Thus, our next study is aimed at determining whether potential mathematical correlation could be explored to calculate actual acetabular anteversion in the standing position, making it possible to more easily and precisely assess the position of the acetabular cup after THA.

## Conclusions

For avoiding the occurrence of adverse events after THA, a simple, accurate, and cheap assessment for cup version is necessary. CL radiography is a potentially eligible method but may present certain errors owing to variations in the PT. For each 1-degree change in the PT, the measurement error can reach 0.76°. However, after correction for the PT using our equation, the measured value can be converted to the approximate anatomical anteversion value under the natural pelvic position, which would be in high agreement with the CT value. Our mathematical algorithm can serve as a reliable method to improve the accuracy of CL radiography.

## Data Availability

The datasets during and/or analysed during the current study available from the corresponding author on reasonable request.

## Abbreviations

CL: radiography Cross-table lateral radiography
VA: Version angle
THA: Total hip arthroplasty
PT: Pelvic tilt
CT: Computed tomography
AP radiography: Anteroposterior radiography
ICC: Intra-class correlation coefficient
